# African Americans’ Perceptions of Adherence to Medications and Lifestyle Changes Prescribed to Treat Hypertension

**DOI:** 10.1177/2158244015623595

**Published:** 2016-01-05

**Authors:** Christina M. Pettey, Jean C. McSweeney, Katharine E. Stewart, Mario A. Cleves, Elvin T. Price, Seongkum Heo, Elaine Souder

**Affiliations:** 1 University of Arkansas for Medical Sciences, Little Rock, AR, USA; 2 North Carolina State University, Raleigh, NC, USA

**Keywords:** hypertension, Blacks, African Americans, qualitative, adherence

## Abstract

More than 80 million Americans have hypertension (HTN), and African Americans (AAs) are disproportionately affected. AAs also have lower rates of adherence to HTN treatment. It is important to understand AAs’ perceptions of adherence to develop effective interventions. The aim of this study is to examine AAs’ perceptions of adherence to medications and lifestyle changes prescribed to treat HTN. In this qualitative study, we used purposive sampling to recruit Southern AAs with HTN aged 21 and older from a free, faith-based clinic. We recorded individual, in-person interviews about perceptions related to adherence to treatment of HTN and analyzed verbatim transcripts using content analysis and constant comparison. We also conducted medical record audits. Twenty-nine AAs participated (52% female, 38% were <50 years of age, 52% had taken anti-HTN medications for ≥5 years). Audits indicated that 65% had uncontrolled HTN during the previous year. Two main themes included causes of HTN and ways to improve blood pressure. Perceived causes of HTN included diet, stress, unhealthy actions, genes, and obesity. Ways to improve HTN included using cultural treatments “passed down,” increasing exercise, reducing stress, and losing weight. Many reported using home remedies to control HTN, including drinking pickle juice. More than half of this sample had uncontrolled HTN. They identified influences of culture on perceptions of adherence including causes and treatment of HTN, and possibly detrimental home remedies. It is imperative that clinicians identify culturally appropriate interventions for this high-risk group.

## Introduction

Although the mortality from cardiovascular disease has declined in the last 10 years, it is still the leading cause of death world-wide (Deaton et al., 2011; World Health Organization, 2015). Hypertension (HTN) is a major contributor to cardiovascular disease and a significant problem in the United States. Thirty-three percent of the adult U.S. population, or approximately 80 million Americans, have HTN (Mozaffarian et al., 2015). African American (AA) adults have the highest rates of HTN in the country (Institute of Medicine, 2011; Mozaffarian et al., 2015). Among people 20 years of age and older in the United States, 45% of AA males and 46% of AA females have HTN, compared with 33% of White males and 30% of White females (Mozaffarian et al., 2015). AAs also have higher HTN-related mortality than other ethnic groups (Institute of Medicine, 2011; Mozaffarian et al., 2015). Furthermore, the incidence of uncontrolled HTN is increasing among AAs (Dennison & Hughes, 2008; Hekler et al., 2008; Mozaffarian et al., 2015). AAs in the South are at greatest risk of HTN and its sequelae (Institute of Medicine, 2008; Mozaffarian et al., 2015) and AAs are also disproportionally affected by poverty, which increases barriers to cardiovascular disease treatment adherence. The average yearly income in the United States for AAs in 2011 was US$32,299 compared with US$55,412 for Whites; 27.6% of AAs were in poverty, compared with 12.8% of Whites (DeNavas-Walt, Proctor, & Smith, 2012). Furthermore, in 2011, 19.5% of AAs were without health insurance coverage, compared with 14.9% of Whites (DeNavas-Walt et al., 2012).

Researchers have suggested that perceptions of HTN treatment adherence contribute to the poorer cardiovascular disease outcomes among AAs (Fongwa et al., 2008; Gatti, Jacobson, Gazmararian, Schmotzer, & Kripalani, 2009). Adherence is defined as behavior that is congruent with prescribed recommendations, including lifestyle changes and medications (Cohen, 2009). Lifestyle recommendations for HTN treatment include diet, exercise, alcohol moderation, stress reduction, and tobacco cessation (Deaton et al., 2011; Mozaffarian et al., 2015). HTN treatment typically includes a combination of these lifestyle recommendations and medications (James et al., 2014; Mozaffarian et al., 2015), yet individuals may institute culturally based home remedies in conjunction with or instead of prescribed treatment, and this can have a profound effect on their treatment outcomes.

Understanding perceptions of adherence to medications and lifestyle changes prescribed to treat HTN may provide the foundation for developing culturally sensitive, tailored interventions to improve adherence for this at-risk population. This qualitative study therefore examined perceptions of adherence to treatment of HTN among poor AAs in Arkansas, the state with the highest stroke mortality in the nation for AA females and the second highest for AA males (Gillum, Kwagyan, & Obisesan, 2011; Mozaffarian et al., 2015).

## Background

Only a few researchers have investigated perceptions of adherence to treatment of HTN among AAs. In one focus group study, 20 low-income, AA females with HTN recruited from a free urban clinic were asked about their knowledge, attitudes, and perceptions regarding HTN and adherence to treatment (Fongwa et al., 2008). Some defined HTN as headache, and others as the heart pumping too hard, causing damage to heart valves, and a buildup of salt, pork, and cholesterol in the blood. Some referred to HTN medications as addictive. Other studies found stress was a perceived cause of HTN, which affected adherence to prescribed treatment (Hekler et al., 2008; Rose, Kim, Dennison, & Hill, 2000; Webb & Gonzalez, 2006). These data suggest a lack of understanding of the causes and treatment of HTN, and these misperceptions may influence treatment adherence.

Culture may also influence adherence to HTN treatment. Religion is a source of support and leadership in the AA community (Allen & Szanton, 2005), reflecting a collectively oriented culture. AAs report a strong commitment to family and community (Webb & Gonzalez, 2006). In one study, 53% reported that family support, usually from a mother or sister, was most important and hearing stories about family and friends who had complications of cardiovascular disease facilitated their decision to seek care (Rose et al., 2000). Thus, experience with family and community members may influence AAs’ decisions to adhere to HTN treatment, but the extent of this influence and the mechanisms of the influence are not well understood.

Culture may influence individuals’ health perceptions and adherence to dietary recommendations. For instance, Horowitz and colleagues assessed AAs’ and Latinos’ knowledge, attitudes, behaviors, and perceptions of diet and HTN in nine focus groups (four AA groups and five Latino groups, *n* = 88); 77% were females, 53% were AA, and 75% were poor (earned <US$15,000; Horowitz, Tuzzio, Rojas, Monteith, & Sisk, 2004). All four AA groups believed that diet contributed to HTN, but many found it difficult to adhere to clinician-recommended diet changes because of cultural preferences for salt, fat, and pork. Some complained of feeling isolated when they tried to follow diets different from the rest of their family and friends. Participants in three of the nine focus groups believed that diet would not negate the need to take medications for HTN and therefore they did not place high importance on adhering to dietary recommendations. Researchers of the study did not identify which of the groups reported those findings (AA or Latino). Interestingly, participants in one AA focus group reported using garlic, herbs, and vinegar to control HTN, in addition to prescribed treatment.

### Adherence to Medications and Lifestyle Changes

AAs consistently have lower rates of adherence to HTN treatment than other groups. Shaya et al. (2009) reported that AAs had lower adherence to HTN treatment (angiotensin converting enzyme inhibitors/hydrochlorothiazides [ACEIs/HCTZs] or ACEI/calcium channel blockers) than Whites (19.69% vs. 36.17%) and other racial/ethnic groups (36.11%) (*p* = .0001; Shaya et al., 2009). An anonymous survey of 244 AAs and Whites with HTN who were patients in two primary care clinics in Ohio found that Whites were significantly more likely than AAs to be engaged in exercise (36% vs. 17%), but following a diet to treat HTN did not differ by race/ethnicity (*p* = .29; Wexler et al., 2008). Research has also shown that gender, self-efficacy, trust in the health care system, medication pill burden, age, level of systolic or diastolic blood pressure (BP), and comorbidities are associated with adherence rates (Elder et al., 2012; Gatti et al., 2009; Irvin et al., 2012; Lagu et al., 2009; Muxfeldt, de Souza, & Salles, 2013; Shaya et al., 2009). Differences in adherence rates may thus be due to differences in cultural perceptions of adherence to treatment of HTN. It is also known that some medications such as beta blockers, ACEIs and angiotensin receptor blockers (ARBs) are not as effective in AAs than in other racial/ethnic groups (Johnson, 2008), and it is possible that AAs have lower rates of adherence to these medications because they are not effective; if the medications do not work well, they may stop taking them. However, when taken in combination with other antihypertensive medications, they are effective. Also, HTN is often an asymptomatic disease and AAs may stop taking medications due to undesirable side effects they did not have previously.

## Sensitizing Framework

The Health Belief Model (HBM) suggests that asymptomatic individuals with HTN may not follow prescribed treatment regimens unless they accept the fact that although they have no symptoms, they do have HTN (perceived susceptibility). They must understand that HTN can lead to complications such as strokes and heart attacks (perceived severity). They also must understand that following prescribed treatment regimens will reduce their risk of complications (perceived benefits), without excessive difficulty such as cost and time commitment or negative side effects (perceived barriers). Cues to action such as pill reminders and handouts may be beneficial to increase health-promoting behaviors. And finally, self-efficacy is necessary for individuals to feel competent enough to attempt health-promoting behaviors such as preparing healthy meals and exercising (Janz, Champion, & Stretcher, 2002).

The HBM is helpful in understanding health promotion behaviors and does include ethnicity as a modifying factor, but it does not strongly address the influence of culture; few studies have addressed culture within ethnicity in the context of the HBM. Culture is thought to play an important role in adherence to prescribed treatments (Fongwa, Evangelista, & Doering, 2006; Fongwa et al., 2008; Gatti et al., 2009), though it is not clear how culture influences adherence among AAs. Therefore, with the addition of culture, the HBM was used as a sensitizing framework to explore and understand AAs’ perceptions of adherence to treatment of HTN.

## Method

### Study Design, Participants, and Setting

Methods have been described previously (Pettey et al., 2015). Briefly, to examine perceptions of adherence to treatment of HTN among AAs, we used a qualitative approach with ethnographic interview techniques. Arkansas ranks among the worst in the nation in age-adjusted cardiovascular disease deaths (Mozaffarian et al., 2015). We used purposive sampling (Streubert & Carpenter, 2011) to recruit patients from a faith-based, urban primary care clinic in Arkansas that serves uninsured, indigent residents aged 21 to 64 years. Patients are seen free of charge and receive free medications and laboratory work, if indicated.

Inclusion criteria were (a) identified as AA by self-report, (b) treated with medications for HTN for at least 6 consecutive months, (c) 21 to 64 years of age, and (d) currently being treated at the clinic. Exclusion criteria were (a) dementia, (b) profound deafness, and (c) severe speech impairment. People seen in the clinic by the author as a provider in the previous 12 months were also excluded. To rule out dementia, the author administered the Short Blessed Test (SBT), which evaluates orientation, attention, and memory, has established reliability and validity, and has been used to screen AAs for dementia (Ball, Bisher, & Birge, 1999; Katzman et al., 1983; Wilkins et al., 2007).

The Clinic Manager of the recruitment site for the study provided full support for the study. A culturally sensitive colorful flyer with the author’s contact information was placed in the main clinic waiting area to inform potential participants about the study, and clinic personnel distributed flyers to patients at check-in. Those who were interested telephoned the author or approached her in person if she was available in the clinic. The author then explained the study, answered questions, obtained initial verbal consent, administered the SBT to rule out dementia, and scheduled an interview at a mutually acceptable time and location. Those who agreed to participate signed and dated two copies of the written consent and Health Insurance Portability and Accountability Act (HIPAA) forms in person prior to their interview. Study participants were informed that they may be interviewed 2 to 3 times and all gave written consent to be recontacted for further interviews. We obtained Institutional Review Board (IRB) approval prior to beginning the study. Recruitment ceased when (a) theoretical saturation was achieved, which was determined by repetition of information so that we ceased to learn anything new from participants (Streubert & Carpenter, 2011) and (b) approximately equal numbers of males and females were recruited. Participants received a US$20 Walmart gift card for the initial interview and a US$5 Walmart gift card for the second interview. No third interviews were conducted.

To ensure confidentiality of the data, the author generated a master list of names, assigned a code number to each, and stored the list in a locked, secure location. Audiotapes, transcriptions of the interviews, and data collection forms were labeled only with the code number and were locked in a separate, secure location. The author collected all data.

### Data Collection

The author collected data through (a) demographic forms, (b) individual face-to-face in-depth interviews, (c) field notes, and (d) medical record audits. The author completed a demographic form with information provided verbally by the participant prior to the initial interview. This form included questions on age, sex, race, level of education, marital status, and of length treatment for HTN. BP was not measured directly but was recorded as high (≥140/90) or normal as documented in the medical record.

Initial individual in-person, semistructured interviews lasted approximately 30 to 60 min and were held at a place of the participants’ choosing. Most participants (73%) elected to be interviewed in a private room at the clinic; the remainder were interviewed in their homes. All interviews followed the same guide and began with, “How did you first learn about your high BP?” a nonthreatening question to stimulate and direct the discussion (Rubin & Rubin, 2012). Probe questions were asked to expand and clarify information (Streubert & Carpenter, 2011).

The author dictated field notes into a recorder immediately after each interview. These field notes included the author’s observations of the appearance and emotional state of the participant and the author’s reactions to the participant’s responses. Field notes enhanced reflection and facilitated the author’s assessment of her potential influence on the research. To ensure rigor and credibility, an experienced qualitative transcriptionist transcribed interviews verbatim. The author verified transcriptions and validated findings through member checking with 12 participants during second interviews. The author conducted all interviews and discussed methodologic decisions with other research team members.

A medical record audit form compatible with REMARK Classic OMR® (1991) software was used to gather data from the clinic record, including confirmation of the diagnosis of HTN, names and doses of all medications ordered, BP readings, and clinician-recommended lifestyle changes. The form was adapted from one used in National Institutes of Health-funded longitudinal study of more than 1,000 AA and White females (McSweeney et al., 2014). Clinic visits for the past 12 months were audited to capture at least two clinic visits with HTN treatment recommendations.

### Data Analysis

All data were reviewed for completeness and validity by running frequencies and scatterplots to look for outliers and incorrectly entered data. Statistics of central tendency and variation were used to describe the sample.

Colaizzi’s (1978) nine-stage framework was used for qualitative analysis of interviews and field notes. Data collection and analysis were concurrent, as is recommended with ethnographic interview techniques, permitting each interview to guide subsequent interviews (Rubin & Rubin, 2012).

Audiotaped interviews and field notes were transcribed verbatim by an experienced qualitative transcriptionist (Colaizzi, 1978). The author checked transcriptions for accuracy while listening to the audio recordings, then entered them into the Ethnograph v6.0 computer program, which was used to organize the data and facilitate retrieval for analysis (Streubert & Carpenter, 2011).

After entering two sets of interviews (two males and two females) into Ethnograph, an initial content analysis was performed, with a line-by-line review of each transcript and identification of key concepts (Colaizzi, 1978). The author carefully read and reread the transcribed interviews to identify major concepts, and these provided the basis for code words. Initial code words were verified by members of the research team and then incorporated into a code book that served as a guide for coding the remaining interviews (Colaizzi, 1978).

Following content analysis and coding of interviews, the author used the technique of constant comparison, an iterative process of comparing and contrasting each datum with all other data to gain conceptual understanding. Data on each topic were compared to identify meanings, similarities, differences, and relationships.

Data were then aggregated and clustered into interrelated units of meaning or categories to develop themes (Colaizzi, 1978). Through this iterative process, AAs’ perceptions of adherence to treatment of HTN and cultural issues were identified. Findings were validated with participants verbally in second interviews, and new findings were incorporated (Colaizzi, 1978).

## Results

### Participant Characteristics

Twenty-nine AAs participated (15 females, 14 males), 18 (62%) were ≥50 years of age, 72% had ≥12th-grade education, and 52% had taken anti-HTN medications for ≥5 years (see Table 1). All participants were poor (<200% of the federal poverty level) and uninsured. Seventeen (65%) had elevated BP on at least one clinic visit (five males, 45%; 12 females, 80%). Medical record audits also revealed that the mean number of anti-HTN medications prescribed for male participants was 1.8, ranging from 1 to 3, and the mean number of anti-HTN medications female participants were prescribed was 2.3, ranging from 1 to 4. Diuretics were prescribed most frequently (73% of both genders), followed by ACEIs (45% of males and 47% of females), and then beta blockers (36% of males and 47% of females). No males (0%) and only two females (13%) had documented health professional recommendations for lifestyle changes. However, interviews indicated that participants had been told to make lifestyle changes by clinicians. The mean number of visits per participant was 3, with a range from 1 to 7.

### Themes

The two main themes were AA perceived causes of HTN and ways to improve BP.

#### Theme 1: Perceived causes of HTN

Participants were asked, “What do you think causes high BP?” Probe questions included, “How does that affect what you do for your high BP?” Perceptions of the causes of HTN were explored because perceived causes of illness may be linked to treatment adherence (Rose et al., 2000). Data were aggregated into four global subthemes: AA dietary traditions, life, health issues, and gaps in knowledge. There were some differences in male and female perceptions (see Table 2).

#### Subtheme 1a: AA dietary traditions

Participants noted both foods and food preparation as causes of HTN and elevated BP. Twenty-six participants identified the Southern AA diet as a major cause of HTN. When one male was asked why he thought HTN ran in his family, he said, “Hummm, I’m not sure, maybe problems with not eating right, bad eating habits passed down.” Many identified sodium and pork in particular as causes of HTN. For instance, one male said, “You can’t eat a lot of salt. You get dizzy, swimming in the head. And pork causes it [BP] to go higher.” Six participants discussed food preparation as a cause of HTN. One male said,

Growing up Black, the diet was something both my parents realized they needed to improve on. I remember my mom was an excellent cook, to season food there was always a piece of salt meat that was cooked in the food … it was the real cause of the high blood. Back then they didn’t call it hypertension, they called it high blood.

This subtheme illustrates how culture may be found within ethnicity as a modifying factor in the HBM.

#### Subtheme 1b: Life

The second most frequently discussed subtheme was life, including stress, unhealthy actions, and running out of medications as causes of HTN. The majority of participants identified stress as a cause of HTN. One male said, “Stress, I got too much stress now. Everybody stresses out but you never know how stress runs your pressure up.” One male participant discussed losing his job as a source of stress and a major cause of his HTN. He said, “Two years ago I lost my job after 20 years of working for an organization I started—sent my BP through the roof.” One woman who had recently been homeless described what she thought caused her to develop HTN: “Stress can cause high BP, your surroundings, when you are angry and bitter all the time.” Another woman said, “I can feel what brings it on too, stress, life.”

Many discussed unhealthy actions as a cause for their HTN, including drinking alcoholic beverages, not following God’s plan, and using illicit drugs. For instance, one male said, “I used to drink alcohol but I quit that. I guess that is what got my pressure up.” Another male said, “I didn’t focus on God and I was doing what the Devil wanted me to do.” One male clearly indicated illicit drugs: “Well I tell you what, cocaine causes your heart to beat faster and your BP to go up.”

Running out of medications was identified by three participants as a cause of their high BP and a reason for nonadherence. They said life sometimes gets in the way of scheduling clinic appointments to obtain refills due to work and family obligations. One male said, “I try to take it [prescribed medication] at the same time every day. Just take it the same time, eat right, and try to remain stress free. When you run out, that is when it [BP] really gets high.” This sub-theme illustrates several perceived barriers to adherence encountered in life for this population as described in the HBM.

#### Subtheme 1c: Health issues

This subtheme included genetics, obesity, and physical causes. One male participant said, “I just figured I know it’s a gene in the family, someone that had it before and it runs in the family.” Another male said, “Ya know, I stopped to think about this. I know I had high BP and they said it was hereditary like diabetes.” Five identified obesity as a cause of HTN. One woman stated, “Not eating right, fat, gaining weight.” We did not collect data on weight, but four of the five who discussed this appeared to be overweight or obese. Three participants identified physical causes, such as a kidney or heart disorder, as contributing to their HTN. This subtheme demonstrates the construct of perceived susceptibility in the HBM; participants described things they thought caused their HTN.

#### Subtheme 1d: Gaps in knowledge

Two participants also identified lack of knowledge as a cause for HTN. One woman said, “I didn’t know that pickles caused your BP to rise. That’s the thing, not knowing, that’s the silent killer right there.”

**Figure 1:**
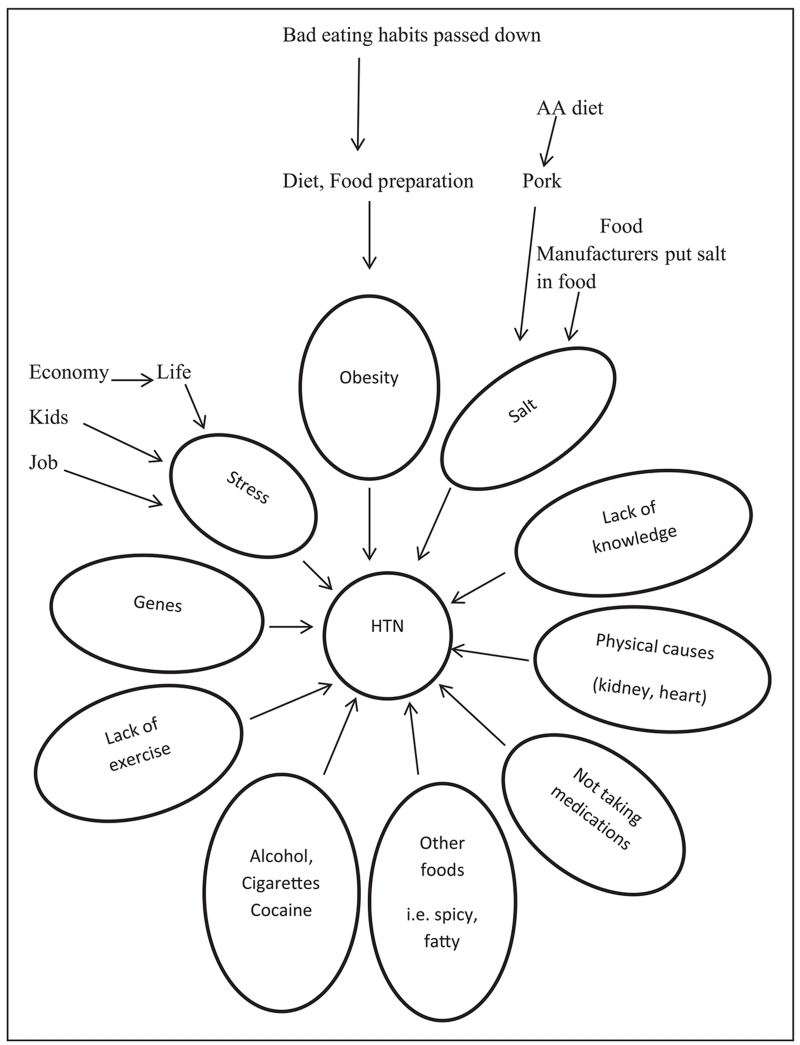
Perceived causes of hypertension (*N* = 29). *Note.* AA = African American; HTN = hypertension.

Figure 1 lists all the causes of HTN identified by participants. It was apparent from the interviews that participants believed these things raised BP but they did not know why.

Interestingly, as shown in Figure 1, the participants discussed lack of exercise as a cause of HTN but did not connect lack of exercise with obesity.

#### Theme 2: Perceived ways to improve BP

Participants were asked, “What do you do for your high BP?” and “How do you decide what things to do for your high BP?” to elicit participants’ perceptions of adherence to HTN treatment. Data were aggregated into two global subthemes: AA culture and necessary changes (see Table 3).

Subtheme 2a: AA culture. When describing what they did to lower BP, participants discussed eating habits and treatments passed down in relation to the AA culture. All participants discussed changing eating habits in an attempt to lower their BP, and 26 reported attempting to lower their salt intake. However, some lacked the knowledge necessary to do so effectively. In fact, six participants reported using seasonings that contained salt as a means to lower their salt intake and thought they were adhering to clinician-recommended dietary changes. For instance, one male said, “I try to watch my salt intake, we use like sea salt.” Another male said, “I try to buy more natural stuff … and it may not have salt and then you can add your own salt to it.” And a third male said, “I really don’t like salt. I use seasoned salt for flavor but I don’t like salt, period.” Others discussed avoiding pork or altering the way they cooked it. For instance, one male said, “I don’t eat pork like I used to, but if we do a pork chop or something we will bake it or broil it and cut it with a little apple cider vinegar before we cook it.”

Treatments passed down from generation to generation included prayer and home remedies. Males discussed prayer and turning to God to lower BP more often than females (six vs. one), and this did not differ significantly by age (the mean age of those reporting prayer was 54 years compared with 48 years for those who did not discuss prayer as a way to lower BP, *p* > .1). For instance, one male said,

I just turn everything over to God and let Him work it out. I mean, let Him be the one to take care of me and do what He says … and I try to read and study my Bible and do what it says and that is what I try to do every day and follow the Word and live by the Word.

Participants identified alternative treatments including mustard, apple cider vinegar, garlic, and pickle juice. The amount of vinegar consumed varied from one capful to ½ cup daily. One woman said, “A lot of people tell me they eat a little mustard, a little vinegar, or a little pickle juice. Pickle juice because of the pickle juice has vinegar in it.” One woman said, “I eat pickles and drink the juice [to lower BP].” Interestingly, one male participant hypothesized why these home remedies were passed down. He said,

My grandfather would tell you about BP, old Black people didn’t go to the hospital and they had to make their own remedies. It has nothing to do with hatred, it had to do with age and there wasn’t no medicine and that’s why they had to come up with their own remedies.

These treatments passed down are further examples of culture within the modifying variable of ethnicity in the HBM.

#### Subtheme 2b: Necessary changes

Participants described doing exercise, taking medicine, reducing stress, avoiding substances, and losing weight to lower BP. All discussed exercise as a means to lower BP; many discussed walking either for exercise or out of necessity and felt that lowered their BP. One male said,

It is good to walk … and do more than go around this building, maybe one day walk one side and the next day walk the other side. You got to get your stuff in your body to move around, you can’t just sit there.

One woman said, “I do a lot of walking, just getting from here to there.” And a male said, “The work I do, I do a lot of walking.” Participants demonstrated self-efficacy found in the HBM when discussing exercise and other lifestyle changes.

#### The majority said that they attempted to be adherent to prescribed medication

Only one male reported that he was not adherent. Several discussed taking medicine as a routine. For instance, one male said, “I normally take it [medication] in the morning. Every morning I go take two pills. It’s like a routine every day.” Another male said, “I always make sure I take them [medications]. I never forget. I won’t ever forget a thing like that. Once the doctor prescribes me, I take it.” Incorporating medications into an existing routine is an example of cues to action in the HBM. Linking taking medication to things they were already doing helped them remember to take them. The majority of participants reported that they did their best to adhere to prescribed medications, but some discussed difficulty in obtaining refills and said they ran out of medications at times.

Many participants discussed reducing stress to lower BP. One male said, “I have to lower my stress, if I don’t I’ll probably kill myself or I will have a heart attack. I get stressed about bills, you know, life. I have to calm down a bit.” This quote is an example of perceived severity in the HBM. Another male said, “You got to calm yourself down for them [medications] to work. You got to sit down and calm down or read a paper, something that will calm your nerves for the BP to work.” One male said, “I try not to get in no stressful situations but you can’t go through life without stressful situations.”

Some discussed avoiding alcohol, tobacco, and illicit drugs to lower their BP. For instance, one male said,

I have cut back big time [on cigarettes]. I probably smoke one and don’t need it and then one day I thumped a whole one away trying to cut them away so I am doing real good. I hadn’t had a cigarette in the past 72 hours.

Another male said, “I used to drink alcohol. I stopped all that.” They knew these unhealthy actions raised BP and therefore tried to stop them to lower their BP. This demonstrates perceived benefits to adherence in the HBM.

Nine discussed losing weight to lower BP. For instance, one male said, “You got to watch your weight … since I got out of the hospital I try to lose.” One woman said, “I am trying to be conscious of how I handle my weight, trying to get some of it off.” Most discussed eating healthy foods such as vegetables, fruit, and turkey, as well as baking instead of frying in an attempt to lose weight. However, few seemed to connect exercise with losing weight. Many reported exercising to lower BP but not to lose weight.

## Discussion

Participants in this study were poor AAs. Many discussed how culturally derived perceptions of the causes and treatment of HTN influenced adherence decisions. The most frequently noted causes of HTN were the Southern AA diet and stress; the most commonly reported ways to improve BP were changing eating habits, exercising, and taking medications. Reducing stress was also a common treatment attempted. Thus, the perceived causes of HTN were linked in many ways to self-treatment instituted by participants and were consistent with findings of other studies of AAs with HTN (Fongwa et al., 2008; Horowitz et al., 2004; Wexler et al., 2008).

The themes identified in this study are consistent with the theoretical framework of the HBM. Participants discussed perceived susceptibility due to family history and other risk factors such as diet. One participant discussed symptoms of dizziness when his BP was elevated. Several participants reported perceived severity when discussing things that cause their BP to rise. Many also voiced perceived benefits related to adhering to medications and lifestyle changes and perceived barriers including obtaining medication refills. Several reported using cues to action such as incorporating medications into a daily routine and many seemed to possess self-efficacy when discussing taking medications and making lifestyle changes. Other studies have had similar findings (Gatti et al., 2009; Schoenthaler, Ogedegbe, & Allegrante, 2009). However, even though the perceptions of participants aligned with the HBM, the majority of them had elevated BP on at least one clinic visit per medical record audits.

AAs have frequently been found to have high rates of uncontrolled HTN (Mozaffarian et al., 2015). More than half of the participants (65%) in this sample (45% of the males and 80% of the females) had elevated BP at one or more of their clinic visits even though they had access to free health care and medications. Similarly, another study found that providing more adults with health insurance coverage through Medicaid did not improve HTN outcomes (Baicker et al., 2013). This illustrates that free health care may not improve HTN outcomes for this population. Quality and accessibility of free services offered to AAs must be ensured.

Medical record audits revealed that only two participants (both female) had documented counseling regarding lifestyle changes, but in interviews, most participants mentioned lifestyle changes that clinicians had recommended. It appeared that clinicians at the clinic were teaching about lifestyle changes but not documenting this. Similarly, another study found that dietary education had been provided to AAs with HTN but the information provided was hard to follow (Horowitz et al., 2004). It is difficult for clinicians to follow up on education and recommended lifestyle changes when they do not know what was taught previously. It is imperative that patients receive instructions they can understand so that they can implement lifestyle changes to lower BP. Dietary changes may be difficult due to cultural preferences and these issues should be explored by clinicians.

Adherence rates are lower for AAs than other groups (Mozaffarian et al., 2015; Shaya et al., 2009) but this sample of AAs said they tried to adhere to medications and necessary lifestyle changes. Participants could have over reported their adherence rates (Burnier, Wuerzner, Struijker-Boudier, & Urquhart, 2013). However, the purpose of this study was not to measure adherence but to explore perceptions of HTN treatment among AAs, a group at high risk for HTN-related complications. Most participants in the study reported that they desired to adhere but some found it difficult. Females were less controlled than males even though they were prescribed more medications. Females may have been less adherent because they had a higher medication pill burden (Shaya et al., 2009). It is also possible that AA females are less adherent due to the “Strong Black Woman” concept; they tend to take care of others and neglect themselves (Watson & Hunter, 2015).

The clinic these AAs attended provides 3 months of medications at a time and patients must remember to telephone for an appointment to obtain a refill 2 weeks before they run out of medications. Even though these participants had access to free clinic visits and medications, some reported running out of medications because they could not schedule an office visit. Free clinics are often booked well in advance and it is difficult to obtain an appointment. This illustrates again that access to free services can be problematic and can be a barrier to adherence to treatment.

Food and stress were commonly perceived causes of HTN, consistent with other studies (Fongwa et al., 2008; Horowitz et al., 2004; Wexler et al., 2008). Interestingly, participants also identified lack of knowledge as a cause of HTN and their reported treatment of HTN demonstrated a lack of knowledge: some home treatments such as drinking pickle juice may have been detrimental to their BP. Although only four participants mentioned using pickle juice to lower their BP, it is likely that others used it as well. Using pickle juice to lower BP could be another example of the influence of culture within the modifying factor of ethnicity in the HBM. We are unaware of any other studies reporting the use of pickle juice to lower BP. More research should be done in this area to find out whether this is a cultural norm or unique to this sample.

Participants also reported consuming vinegar with their prescribed medications, but it is not known how vinegar and medications interact. Another study also identified vinegar as a home remedy used by AAs to treat HTN (Horowitz et al., 2004). It is biologically plausible that vinegar could lower BP. For example, vinegar could alter the pH in salt sensitive hypertensive patients and lower BP. This observation is supported by the fact that genetic variations in the pathways that regulate renal acid base status have been shown to cause both HTN and hypotension (Carey et al., 2012; Gröger et al., 2012; Nabel, 2003; Riepe, 2009). In fact, vinegar has been shown to lower BP in rats (Matsushima et al., 2014) but there are no known studies in humans.

Many in this sample knew that they needed to lower their salt intake and lose weight, but they did not know how to do so. This is consistent with the findings of other studies (Fongwa et al., 2008; Horowitz et al., 2004). Because AAs are more salt sensitive than other groups, research has shown that a daily reduction of 1,200 mg of sodium per person could reduce cardiovascular disease among AAs by 15% as compared with 10% for Whites (Bibbins-Domingo et al., 2010).

Some gender differences were found in the ways that AAs treated HTN. Males reported using prayer more often than females to lower BP, and this finding did not differ by age. In a recent qualitative study that examined how 54 AA males and females use religion (Hamilton, Moore, Johnson, & Koenig, 2013), males were more likely to view God as a healer, while females were more likely to view God as a protector. This could explain why males in the current study turned to God more for healing than females did.

Interestingly, several participants reported learning about HTN from family members. As members of a collectively oriented culture, family members of AAs play an important role in health decisions. Thus, clinicians must educate both patients and their families. Clinicians cannot assume that patients will receive or remember instructions. Therefore, they must provide both patients and family members all necessary education, including common medication side effects and implications of nonadherence to treatment. Telephone follow-up may be an inexpensive way to promote adherence and can be done by nursing staff. This would also be a good time to elicit information about any side effects patients may be experiencing and find out whether they are adhering to treatment recommendations.

### Limitations

There were some limitations to this study. Workers at the free clinic could not locate medical records for three participants. Therefore, medical record data cover 26 of the 29 participants. Also, the study was conducted with a small, nonrandomized sample of participants from one faith-based clinic and explored only perceptions of poor AAs receiving free care. However, the goal was to explore personal experiences and perceptions of members of the AA culture. The data add to our knowledge of this high-risk group and may be useful to develop culturally appropriate interventions to reduce cardiovascular disease among AAs.

It may be a limitation that the author was not a member of the same cultural group as the participants. Also, data on perceptions were limited to what participants divulged, based on the assumption that participants would share their perceptions of adherence to prescribed treatments. However, the author has worked as a family nurse practitioner for more than 10 years diagnosing and treating HTN among AAs, and her experience in discussing HTN and related issues with AA patients aided in data collection.

## Conclusion

Perceptions of the causes of HTN may influence self-treatments attempted by individuals. For instance, in this sample, many discussed diet as a cause of HTN and all attempted to make dietary changes to lower BP. Clinicians should query patients about their perceptions of the causes of HTN and learn about the ways they have attempted to treat their HTN. Lack of knowledge was an important finding in this sample and was a barrier to adherence to lifestyle changes. Participants said that they had been told to avoid salt and lose weight but they did not know how. They also did not know the potential detrimental effects of some home remedies. These findings suggest that home treatments should be explored, and patients should be educated about appropriate ways to reduce BP during clinic visits. It is not enough to tell patients to lower their salt intake and eat healthy; clinicians must give specific instructions about what they should and should not do. Clinicians could also ask a few quick questions to elicit information regarding patients’ level of knowledge. For instance, they could ask, “What do you do to lower your BP?” and “What do you use to season food?” Those questions would only take 1 to 2 min and the clinician could quickly glean information about home remedies, level of knowledge of lifestyle recommendations, and adherence to medications. Medication adherence could specifically be assessed using the four- or eight-item Morisky Medication Adherence Scale, which is quick and easy to administer (Krousel-Wood et al., 2011; Morisky, Ang, Krousel-Wood, & Ward, 2008). It is also important for patients to be able to obtain clinic appointments so refills can be prescribed in a timely manner to eliminate that barrier to adherence. Future research should identify culturally sensitive interventions to improve HTN treatment outcomes for this high-risk group.

## Figures and Tables

**Table 1:** Demographic Characteristics of Participants, *n* (%).

Demographics	Total sample (*N* = 29)	Male (*n* = 14)	Female (*n* = 15)	*p* value^a^
Age				
<50	11 (37.9)	5 (35.7)	6 (40.0)	1.0000
≥50	18 (62.1)	9 (64.3)	9 (60.0)	
Education				
<12 years	8 (27.6)	3 (21.4)	5 (33.3)	0.3710
High school graduate	8 (27.6)	6 (42.9)	2 (13.3)	
Some college	9 (31.0)	4 (28.6)	5 (33.3)	
College graduate	4 (13.8)	1 (7.1)	3 (20.0)	
Marital status				
Never married	8 (27.6)	3 (21.4)	5 (33.3)	0.0961
Married	8 (27.6)	3 (21.4)	5 (33.3)	
Divorced	10 (34.5)	8 (57.1)	2 (13.3)	
Widowed	2 (6.9)	0 (0.0)	2 (13.3)	
Declined	1 (3.4)	0 (0.0)	1 (6.7)	
Length of HTN treatment:				
6-12 months	4 (13.8)	1 (7.1)	3 (20.0)	0.6015
1-5 years	10 (34.5)	6 (42.9)	4 (26.7)	
5-10 years	6 (20.7)	2 (14.3)	4 (26.7)	
>10 years	9 (31.0)	5 (35.7)	4 (26.7)	

*Note.* HTN = hypertension.

a Fisher’s exact test.

**Table 2:** Theme 1: Perceived Causes of Hypertension (*N* = 29).

Subtheme	Factors	Raw data quotes
1a: African American dietary traditions	Diet (*n* = 26)	Pork, that is what it is about Black people, even though it is white meat there is something about the way it is cured; it has a lot of salt.
		Growing up Black … pork was the least expensive food but parents stretching the dollar and realized the pork was killing their family.
	Food preparation (*n* = 6)	It was how we were brought up and how we eat, the fried food.
1b: Life	Stress (*n* = 22)	Stress, I get angry.
		When I get upset, stressed.
		Kids will run your pressure up, I mean for real.
		Getting overworked.
	Unhealthy actions (*n* = 16)	Using cocaine.
		A lot of bad habits bring it on like smoking or drinking.
		Lack of exercise.
	Running out of medications (*n* = 3)	When you run out, that is when it really gets high.
1c: Health issues	Genetics (*n* = 18)	It’s just in our DNA.
		It’s a genetic thing.
		It runs through the generations.
		I guess it’s in your genes.
		It can be hereditary.
	Obesity (*n* = 5)	Weight, at that time (of diagnosis) I was overweight.
		You are going to get overweight and it is going to mess with your heart, your blood pressure.
		Weight, and the fact that your heart has to beat harder.
	Physical causes (*n* = 3)	I heard that it had something to do with the kidney or something the kidney was doing.
		In my case a blockage in your heart, your heart had to pump harder.
1d: Gaps in knowledge	Lack of knowledge (*n* = 2)	I didn’t know what caused high blood pressure. If I had known, I would have been taking it seriously.

**Table 3:** Theme 2: Perceived Ways to Improve Blood Pressure (*N* = 29).

Subtheme	Factors	Raw data quotes
2a: African American culture	Eating habits (*n* = 29)	That pork, we love pork. Not saying that Whites don’t eat pork but we love pork. The small neck bone, bacon I eat a lot of bacon but you got to cut back. You take this medicine and then you eat pork, the medicine is not going to work.
		I try to avoid salt but I use a little salt but I don’t use that much. I try to stay as natural as I can. Sometimes I use something in a can but I wash it or boil the salt out of it.
		… they told me about diet and changing things I have eaten in the past, a lot of it is culture.
	Treatments passed down (*n* = 20)	Vinegar works, it does work. I take some myself. I take a capful every morning with the pressure medicine. I don’t know what the vinegar has to do with it but it’s a home remedy but it does work.
		I just turn everything over to God and let Him work it out. Pickle juice.
2b: Necessary changes	Exercise (*n* = 29)	I’ve tried to do more walking.
		I’ve been exercising, walking 3 times a week. That exercise makes a big difference.
	Taking medicine (*n* = 28)	Ya know you have to take your medicine if not you going to have a stroke or a heart attack.
		They put me on medicine so I just take my medicine every day.
	Reducing stress (*n* = 21)	That is one of the things we could do is to avoid stress, that would really help us all out. I know I don’t have any money. Sometimes there are things that I want and I can’t buy them but the money I get but by the time you pay your rent you have nothing left. But I can’t let that stress me out.
		I refuse to get into stressful situations or let anything stress me out. So people could control their blood pressure because it is not just salt it is stress too … if you refuse to get into stressful situations like that then it can maintain ourselves because I refuse to let anything or anybody stress me and my blood pressure.
	Avoiding substances (*n* = 17)	I don’t drink alcohol anymore and I don’t smoke.
		I used to smoke but I haven’t had a cigarette in about 20 years.
	Losing weight (*n* = 9)	I’ve got to keep my weight down.

## References

[R1] Allen J, Szanton S (2005). Gender, ethnicity, and cardiovascular disease. The Journal of Cardiovascular Nursing.

[R2] Baicker K, Taubman SL, Allen HL, Bernstein M, Gruber JH, Newhouse JP, Finkelstein AN (2013). The Oregon experiment—Effects of Medicaid on clinical outcomes. The New England Journal of Medicine.

[R3] Ball LJ, Bisher GB, Birge SJ (1999). A simple test of central processing speed: An extension of the short blessed test. Journal of the American Geriatrics Society.

[R4] Bibbins-Domingo K, Chertow GM, Coxson PG, Moran A, Lightwood JM, Pletcher MJ, Goldman L (2010). Projected effect of dietary salt reductions on future cardiovascular disease. The New England Journal of Medicine.

[R5] Burnier M, Wuerzner G, Struijker-Boudier H, Urquhart J (2013). Measuring, analyzing, and managing drug adherence in resistant hypertension. Hypertension.

[R6] Carey RM, Schoeffel CD, Gildea JJ, Jones JE, McGrath HE, Gordon LN, Felder RA (2012). Salt sensitivity of blood pressure is associated with polymorphisms in the sodium-bicarbonate cotransporter. Hypertension.

[R7] Cohen SM (2009). Concept analysis of adherence in the context of cardiovascular risk reduction. Nursing Forum.

[R8] Colaizzi PF, Valle R, King M (1978). Psychological research as the phenomenolo-gist views it. Existential phenomenological alternative for psychology.

[R9] Deaton C, Froelicher E, Sivarajan Wu LH, Ho C, Shishani K, Jaarsma T (2011). The global burden of cardiovascular disease. European Journal of Cardiovascular Nursing.

[R10] DeNavas-Walt C, Proctor BD, Smith JC (2012). US census bureau, current population reports, P60-243 income, poverty, and health insurance coverage in the United States: 2011.

[R11] Dennison CR, Hughes S (2008). Progress in prevention: Raising the bar to lower blood pressure: Key steps to improve blood pressure control rates. The Journal of Cardiovascular Nursing.

[R12] Elder K, Ramamonjiarivelo Z, Wiltshire J, Piper C, Horn WS, Gilbert KL, Allison J (2012). Trust, medication adherence, and hypertension control in southern African American men. American Journal of Public Health.

[R13] Fongwa MN, Evangelista LS, Doering LV (2006). Adherence to treatment factors in hypertensive African American women. The Journal of Cardiovascular Nursing.

[R14] Fongwa MN, Evangelista LS, Hays RD, Martins DS, Elashoff D, Cowan MJ, Morisky DE (2008). Adherence treatment factors in hypertensive African American women. Vascular Health & Risk Management.

[R15] Gatti ME, Jacobson KL, Gazmararian JA, Schmotzer B, Kripalani S (2009). Relationships between beliefs about medications and adherence. American Journal of Health-System Pharmacy.

[R16] Gillum RF, Kwagyan J, Obisesan TO (2011). Ethnic and geographic variation in stroke mortality trends. Stroke.

[R17] Gröger N, Vitzthum H, Fröhlich H, Krüger M, Ehmke H, Braun T, Boettger T (2012). Targeted mutation of SLC4A5 induces arterial hypertension and renal metabolic acidosis. Human Molecular Genetics.

[R18] Hamilton JB, Moore AD, Johnson KA, Koenig HG (2013). Reading the Bible for guidance, comfort, and strength during stressful life events. Nursing Research.

[R19] Hekler EB, Lambert J, Leventhal E, Leventhal H, Jahn E, Contrada RJ (2008). Commonsense illness beliefs, adherence behaviors, and hypertension control among African Americans. Journal of Behavioral Medicine.

[R20] Horowitz CR, Tuzzio L, Rojas M, Monteith SA, Sisk JE (2004). How do urban African Americans and Latinos view the influence of diet on hypertension?. Journal of Health Care for the Poor and Underserved.

[R21] Institute of Medicine (2008). Challenges and success in reducing health disparities: Workshop summary.

[R22] Institute of Medicine (2011). Leading health indicators for healthy people 2020: Letter report.

[R23] Irvin MR, Shimbo D, Mann DM, Reynolds K, Krousel-Wood M, Limdi NA, Muntner P (2012). Prevalence and correlates of low medication adherence in apparent treatment-resistant hypertension. Journal of Clinical Hypertension.

[R24] James PA, Oparil S, Carter BL, Cushman WC, Dennison-Himmelfarb C, Handler J, Ortiz E (2014). 2014 evidence-based guideline for the management of high blood pressure in adults: Report from the panel members appointed to the Eighth Joint National Committee (JNC 8). Journal of the American Medical Association.

[R25] Janz NK, Champion VL, Stretcher VJ, Glanz K, Rimer BK, Lewis FM (2002). The health belief model. Health behavior and health education: Theory, research, and practice.

[R26] Johnson JA (2008). Ethnic differences in cardiovascular drug response: Potential contribution of pharmacogenetics. Circulation.

[R27] Katzman R, Brown T, Fuld P, Peck A, Schechter R, Schimmel H (1983). Validation of a short orientation-memory-concentration test of cognitive impairment. American Journal of Psychiatry.

[R28] Krousel-Wood M, Joyce C, Holt E, Muntner P, Webber LS, Morisky DE, Re RN (2011). Predictors of decline in medication adherence: Results from the cohort study of medication adherence among older adults. Hypertension.

[R29] Lagu T, Weiner MG, Eachus S, Tang SS, Schwartz JS, Turner BJ (2009). Effect of patient comorbidities on filling of antihypertensive prescriptions. The American Journal of Managed Care.

[R30] Matsushima A, Furuuchi R, Shirai M, Nagai S, Yokoyama T, Nishida H, Hirayama M (2014). Effects of acute and chronic boysenberry intake on blood pressure and endothelial function in spontaneous hypertensive rats. Journal of Nutritional Science and Vitaminology.

[R31] McSweeney J, Cleves MA, Fischer EP, Moser DK, Wei J, Pettey C, Armbya N (2014). Predicting coronary heart disease events in women. Journal of Cardiovascular Nursing.

[R32] Morisky DE, Ang A, Krousel-Wood M, Ward HJ (2008). Predictive validity of a medication adherence measure in an out-patient setting. Journal of Clinical Hypertension (Greenwich, Connecticut).

[R33] Mozaffarian D, Benjamin EJ, Go AS, Arnett DK, Blaha MJ, Cushman M, Turner MB (2015). Heart disease and stroke Statistics—2015 update: A report from the American Heart Association. Circulation.

[R34] Muxfeldt ES, de Souza F, Salles GF (2013). Resistant hypertension: A practical clinical approach. Journal of Human Hypertension.

[R35] Nabel EG (2003). Genomic medicine: Cardiovascular disease. The New England Journal of Medicine.

[R36] Pettey CM, McSweeney JC, Stewart KE, Price ET, Cleves MA, Heo S, Souder E (2015). Perceptions of family history and genetic testing and feasibility of pedigree development among African Americans with hypertension. European Journal of Cardiovascular Nursing.

[R37] REMARK Classic OMR (1991).

[R38] Riepe FG (2009). Clinical and molecular features of type 1 pseudohypoaldosteronism. Hormone Research.

[R39] Rose LE, Kim MT, Dennison CR, Hill MN (2000). The contexts of adherence for African Americans with high blood pressure. Journal of Advanced Nursing.

[R40] Rubin HJ, Rubin IS (2012). Qualitative interviewing: The art of hearing data.

[R41] Schoenthaler A, Ogedegbe G, Allegrante JP (2009). Self-efficacy mediates the relationship between depressive symptoms and medication adherence among hypertensive African Americans. Health Education & Behavior.

[R42] Shaya FT, Du D, Gbarayor CM, Frech-Tamas F, Lau H, Weir MR (2009). Predictors of compliance with antihypertensive therapy in a high-risk Medicaid population. Journal of the National Medical Association.

[R43] Streubert HJ, Carpenter DR (2011). Qualitative research in nursing: Advancing the humanistic imperative.

[R44] Watson NN, Hunter CD (2015). I had to be strong: Tensions in the strong black woman schema. Journal of Black Psychology.

[R45] Webb M, Gonzalez L (2006). The burden of hypertension: Mental representations of African American women. Issues in Mental Health Nursing.

[R46] Wexler R, Feldman D, Larson D, Sinnott LT, Jones LA, Miner J (2008). Adoption of exercise and readiness to change differ between whites and African-Americans with hypertension: A report from the Ohio State University Primary Care Practice-Based Research Network (OSU-PCPBRN). The Journal of the American Board of Family Medicine.

[R47] Wilkins CH, Wilkins KL, Meisel M, Depke M, Williams J, Edwards DF (2007). Dementia undiagnosed in poor older adults with functional impairment. Journal of the American Geriatrics Society.

[R48] World Health Organization (2015). Cardiovascular diseases.

